# Applying psychological theories to evidence-based clinical practice: identifying factors predictive of placing preventive fissure sealants

**DOI:** 10.1186/1748-5908-5-25

**Published:** 2010-04-08

**Authors:** Debbie Bonetti, Marie Johnston, Jan E Clarkson, Jeremy Grimshaw, Nigel B Pitts, Martin Eccles, Nick Steen, Ruth Thomas, Graeme Maclennan, Liz Glidewell, Anne Walker

**Affiliations:** 1 Dental Health Services Research Unit, University of Dundee, Mackenzie Building, Kirsty Semple Way, Dundee DD2 4BF, UK; 2 College of Life Sciences and Medicine, Kings College, University of Aberdeen, Old Aberdeen, Scotland AB24 2UB, UK; 3 Clinical Epidemiology Programme, Ottawa Health Research Institute and Department of Medicine, University of Ottawa, 1053 Carling Avenue, Administration Building, Room 2-017, Ottawa ON K1Y 4E9, Canada; 4 Institute of Health and Society, 21 Claremont Place, Newcastle University, Newcastle upon Tyne, NE2 4AA, UK; 5 Health Services Research Unit, University of Aberdeen, 3rd Floor, Health Sciences Building, Foresterhill, Aberdeen, AB25 2ZD UK; 6 Leeds Institute of Health Sciences, Charles Thackrah Building, University of Leeds, 101 Clarendon Road, Leeds LS2 9LJ, UK

## Abstract

**Background:**

Psychological models are used to understand and predict behaviour in a wide range of settings, but have not been consistently applied to health professional behaviours, and the contribution of differing theories is not clear. This study explored the usefulness of a range of models to predict an evidence-based behaviour -- the placing of fissure sealants.

**Methods:**

Measures were collected by postal questionnaire from a random sample of general dental practitioners (GDPs) in Scotland. Outcomes were behavioural simulation (scenario decision-making), and behavioural intention. Predictor variables were from the Theory of Planned Behaviour (TPB), Social Cognitive Theory (SCT), Common Sense Self-regulation Model (CS-SRM), Operant Learning Theory (OLT), Implementation Intention (II), Stage Model, and knowledge (a non-theoretical construct). Multiple regression analysis was used to examine the predictive value of each theoretical model individually. Significant constructs from all theories were then entered into a 'cross theory' stepwise regression analysis to investigate their combined predictive value

**Results:**

Behavioural simulation - theory level variance explained was: TPB 31%; SCT 29%; II 7%; OLT 30%. Neither CS-SRM nor stage explained significant variance. In the cross theory analysis, habit (OLT), timeline acute (CS-SRM), and outcome expectancy (SCT) entered the equation, together explaining 38% of the variance. Behavioural intention - theory level variance explained was: TPB 30%; SCT 24%; OLT 58%, CS-SRM 27%. GDPs in the action stage had significantly higher intention to place fissure sealants. In the cross theory analysis, habit (OLT) and attitude (TPB) entered the equation, together explaining 68% of the variance in intention.

**Summary:**

The study provides evidence that psychological models can be useful in understanding and predicting clinical behaviour. Taking a theory-based approach enables the creation of a replicable methodology for identifying factors that may predict clinical behaviour and so provide possible targets for knowledge translation interventions. Results suggest that more evidence-based behaviour may be achieved by influencing beliefs about the positive outcomes of placing fissure sealants and building a habit of placing them as part of patient management. However a number of conceptual and methodological challenges remain.

## Background

Dental decay is the most common chronic disease of childhood. In addition to the pain involved, there can be an impact on the children's ability to eat, sleep, and learn, as well as on their emotional well-being and self esteem [1-4]. There is evidence that the prevalence of dental caries in children in Scotland is a significant clinical problem, and that most children are at risk of developing the disease [5]. There is considerable evidence regarding the effectiveness of preventive treatments, and in particular, preventive fissure sealants (PFS). Fissures, particularly deep fissures in the biting surface of teeth are very difficult to clean, and so tend to accumulate debris that leads to the development of caries. The evidence is that sealing fissures in healthy teeth with a plastic coating makes the development of caries much less likely. A Cochrane systematic review [6] found that PFS, relative to no treatment, reduced decay by 86% after 12 months. PFS treatment for children at risk of caries is supported by The American Academy of Paediatric Dentistry, The European Academy of Paediatric Dentistry, and The British Society of Paediatric Dentistry [7-9]. Despite this support, and that PFS application is inexpensive, easy to do, and long-lasting, fewer than 20% of 11 year olds living in Scotland had their first molars sealed at the time of this study [10].

Implementation research, the scientific study of methods to promote the uptake of research findings, includes the development and testing of interventions that enable healthcare professionals to use research findings more effectively [11-13]. However, currently there is little information to guide the choice, or allow the optimisation of the components of such complex interventions when they are introduced into routine care settings [13,14].

Literature reviews suggest that the main problem in this area may be a lack of understanding or description of the mechanism by which these interventions are achieving their effect [15-17]. Since implementing guidelines often require clinicians to change their behaviour, it may be helpful to base implementation interventions on explanatory models explicitly concerned with behaviour change. Many psychological models explain behaviour in terms of predictive beliefs that can be influenced, as well as methods for measuring and influencing them. In effect, they provide a means of focusing the design of an intervention and include an explanation of how it will work. Some evidence exists that support the application of psychological theories to clinical behaviour, but this evidence tends to be limited to one theory or one group of models [*e.g.*, [18,19].

This study, one part of a larger project [20-22], used a number of psychological theories to explore factors associated with the placing of PFS. Factors were drawn from the Theory of Planned Behaviour (TPB) [23,24], Social Cognitive Theory (SCT) [25,26], Implementation Intention (II) [27], Operant Learning Theory (OLT) [28]http://www.bfskinner.org/BFSkinner/Home.html, Common Sense Self-regulation Model (CS-SRM) [29,30], and an adaptation of Stage Models [31,32]. These specific theories, described in detail elsewhere [20], were chosen because they have all been rigorously evaluated in other settings, they all explain behaviour in terms of factors that are amenable to change, and they vary in their emphasis.

At the time of this study, the placement of PFS in Scotland came under a general capitation fee, which meant that there was no data available on the number of PFS actually placed. This meant that it was not possible to explicitly assess this behaviour (see Additional File 1). Two proxy outcomes were included in this analysis. One outcome measure (behavioural simulation) used decisions made in response to written clinical scenarios -- a common means of testing clinical decision-making in medical and dental education. There is also some evidence that scenario-based decision-making is significantly related to actual behaviour [22]. The second outcome was a theoretically derived measure, behavioural intention, because there is also evidence supporting intention as a consistent predictor of subsequent behaviour [16,18,23].

The aim of this study was to identify factors, derived from these psychological models, associated with the decision to place a PFS in six to sixteen year old patients.

## Methods

### Design and participants

The design was a predictive study with theoretical variables and outcomes (behavioural simulation and intention) measured by a single postal questionnaire.

A random sample of 450 general dental practitioners (GDPs) from Scotland were selected from the Scottish Dental Practice Board list by a statistician using a list of random sampling numbers. Eligible participants were GDPs in Scotland who had not been randomly selected to be invited to participate in a previous survey [21] that was part of the larger project [20].

### Predictor measures

Theoretically derived measures were developed following the operationalisation protocols of Ajzen [23,24], Bandura [25,26], Armitage and Conner [33], M Conner and Sparks [34], Moss-Morris [30], Francis *et al*. [35], Blackman [28] and Weinstein [31,32]. The questions were informed by a preliminary, qualitative study with 29 GDPs in Scotland who took part in a semi-structured interview of up to 40 minutes as recommended for the TPB. The interviews used standard elicitation methods and covered the views and experiences about the use of PFS in the management of caries in six to sixteen year old patients. Responses were used, in conjunction with the operationalisation literature (above), to create the questions measuring theoretical constructs. Five knowledge questions were developed by the study team based on areas of good evidence around the use of PFS. Table 1 provides a summary of the predictor measures used in this study (see also [20]); the instrument and its index are available as Additional Files 2 and 3. Unless otherwise stated, all questions were rated on a seven-point scale from 'strongly disagree' to 'strongly agree'.

**Table 1:** Summary of the predictive measures used in the PRIME study investigating beliefs associated with the placing of preventive fissure sealants (PFS)

**Theory of Planned Behaviour **[23]	
Variables (number of items)	Example Item(s)
Behavioural intention (3)	I intend to place FS as a primary part of managing caries in six to sixteen year old patients.
Attitude Direct (2); Indirect^a ^(7) behavioural beliefs (bb) multiplied by 7 outcome evaluations (oe). The score was the mean of the summed multiplicatives.)	D: In general, the possible harm caused by placing PFS is outweighed by its benefits; I: In general, placing a PFS effectively reduces caries risk **x **effectively reducing caries risk is (un/important).
Subjective Norm^b^ Indirect (3) normative beliefs (nb) multiplied by 3 motivation to comply items (mtc). The score was the mean of the summed multiplicatives).	I feel under pressure from the Dental Practice Board to place PFS (nb) **x **How motivated are you to do what the Dental Practice Board thinks you should (mtc: very much/not at all).
Perceived Behavioural Control Direct (5); Indirect/power (10)^c^	D: It is entirely up to me whether I place PFSs; I: I find it difficult to decide in favour of placing a PFS if the patient is a poor attender.
**Social Cognitive Theory **[25,26]	
Risk Perception (6)	It is highly likely that children with medium to high risk of caries will be worse off if I do not place PFS.
Outcome Expectancies Self (2 × 2), Behaviour (7 × 7). The score was the mean of the summed multiplicatives.	S: If I place PFS, then I will think of myself as a caring dentist **x **Thinking of myself as a caring dentist is (Un/Important). B: See Attitude TPB
Self Efficacy General: Generalized Self-Efficacy Scale (Schwarzer, 1992) (10: 4-point scale, not at all true/exactly true); Specific (12)	General: I can always manage to solve difficult problems if I try hard enough. Specific: How confident are you that you can effectively place a PFS in a six to sixteen yr old if the child has poor oral hygiene.
**Implementation intentions **[27]	
Action planning (1)	Currently, my standard method of managing caries does not primarily include placing a PFS.
**Operant conditioning **[28]	
Anticipated consequences (6) Mean	If I routinely place PFS then on balance, my life will be easier in the long run.
Evidence of habit (2) Mean	When I see a patient, I automatically consider placing a PFS.
Experienced (rewarding and punishing) consequences (4): more likely to PFS (score = 1); less likely (score = -1); unchanged/not sure/never occurred (score = 0)). Scores were summed.	Think about the last time you decided to place a PFS in a six to sixteen year old patient and felt pleased that you had done so. Do you think the result of this episode has made you...
**Self-regulation model**^d ^[29,30]	
Perceived identity (3)	Caries is a condition with symptoms generally of an intense nature.
Perceived cause (5)	Caries is caused by poor oral hygiene.
Perceived controllability (7)	What the patient does can determine whether caries reverses or progresses, What I do can determine whether the patient's caries reverses.
Perceived duration (4)	Caries is a condition which is likely to be permanent rather than temporary.
Perceived consequences (4)	Caries does not have much effect on a patient's life.
Coherence (2)	I have a clear picture or understanding of caries.
Emotional response (4)	Seeing patients with caries does not worry me.
**Stage **[31,32]	
Current stage of change. A single statement is ticked to indicate the behavioural stage	Which of these sentences most characterises you at the moment? Unmotivated (3): I have not yet thought about changing the number of PFS I place. Motivated (2): I have decided that I will place more/less PFS. Action (2): I have already done something about increasing/decreasing the number of PFS I place.
**Other measures**	
Knowledge (5) (True/False/Not Sure)	PFS are recommended for routine use with high-risk children.
Demographic	gender, time qualified, number of other dentists in practice, trainer status, hours per week, list size, if the practice employs hygienists.

^a^All indirect measures consist of specific belief items identified in the preliminary study as salient to placing PFS.

^b^These individuals and groups were identified in the preliminary study as influential in the decision to place a PFS

^c^An indirect measure of perceived behavioural control usually would be the sum of a set of multiplicatives (control beliefs x power of each belief to inhibit/enhance behaviour). However, the preliminary study demonstrated that it proved problematic to ask clinicians meaningful questions which used the word 'control' as clinicians tended to describe themselves as having complete control over the final decision to perform the behaviour. Support for measuring perceived behavioural control using only questions as to the ease or difficulty of performing the outcome behaviour was derived from a metanalysis which suggested that perceived ease/difficulty items were sensitive predictors of behavioural intention and behaviour [24].

^d ^Illness representation measures were derived from the Revised Illness Perception Questionnaire [30]

### Outcome measures

#### Behavioural simulation

Key elements that may influence GDPs' decisions to place PFS were identified from the literature (including the SIGN guideline 47 [5] recommendations), expert opinion of the clinical members of the research team, and the initial interviews with 29 GDPs. These elements were categorized into: clinical elements (standard of oral hygiene, clinically detectable caries, unrestored enamel lesions, sugar consumption, number of restorations already present, use of fluoride supplements (toothpaste, tablets), time since last seen); dentist elements (responsiveness to parental pressure, busy clinic, knowledge of patient/patient's family); and patient elements (age, irregular/regular attenders, treatment phobia, parent' desire (does/doesn't want PFS placed), social class, uncooperative). Six clinical scenarios were constructed by randomly choosing six to eight of these elements to describe a situation of patients presenting in primary care. The scenarios were piloted with six dentists and one dental hygienist.

Respondents were asked to decide whether they would place a PFS (score = 1) or would not place a PFS (score = 0). Decisions in favour were summed to create a total score out of a possible maximum of six. In all scenarios, the decision to place a PFS would be following evidence-based practice.

#### Behavioural intention

Three items assessed intention to place PFS: 'I aim to place PFS as part of six to sixteen year old patient management';' I have in mind to place PFS when I see six to sixteen yr olds'; 'I intend to place PFS as a primary part of managing caries in six to sixteen year old patients'. The mean score of the three responses were scaled so that higher scores reflected stronger intention to place a PFS.

### Procedure

The randomly selected dentists were sent an invitation pack (letter of invitation, questionnaire consisting of psychological and demographic measures and a consent form to allow access to their fee claims data, as well as a reply-paid envelope). Three postal reminders were sent to non-responders at two, four, and six weeks after the first mailing.

### Sample size and statistical analysis

The target sample size of 200 was based on a recommendation by Green [36] to have a minimum of 162 subjects when undertaking multiple regression analysis with 14 predictor variables.

Data were analysed using SPSS Statistics 17.0 [37]. Missing data for each item were replaced with the individual's mean over all the items of that measure, providing only two or less items from the measure were missing. The internal consistency of the measures was tested using Cronbach's alpha. If this was less than 0.6, then questionnaire items were removed from each measure to achieve the highest Cronbach's alpha possible. For two question constructs, a correlation coefficient of 0.25 was used as a cut off. The relationship between predictive and outcome variables were examined within the structure of each of the theories, using Pearson correlations and ANOVA (for the stage model categories).

Multiple regression analyses were then used to examine the predictive value of each theoretical model separately (the 'theory-level' analysis). Finally, all significantly predictive variables (p < 0.05), regardless of theoretical origin, were entered into a stepwise regression analysis to investigate their combined predictive value (the 'cross-theory' analysis).

### Ethics approval

The study was approved by the UK South East Multi-Centre Research Ethics Committee.

## Results

Of the 450 GDPs approached, 43 were ineligible (moved practice, retired, deceased). There were 120/407 (29%) respondents who agreed to participate. Sixty-nine were male (58%), they had been qualified for a mean (SD) of 18.77 (9.3) years, they had a median (inter-quartile range (IQR)) list size of 4,500 (2,575 to 7,250); 12 (10%) were trainers. There was an average of one dental hygienist per practice, and GDPs worked on average 8.57 (SD = 2.14) half-day sessions per week.

The representativeness of the study participants was examined by comparing their demographics with the available demographics of the 2006/2007 Management Information Dental Accounting System database, which shows 60% of dentists in Scotland are male and have been qualified on average for 18 years (this was calculated from the available information of: average age = 41/average age qualified = 23).

### Relationship between the two outcome measures

The two outcome measures, behavioural simulation and behavioural intention, were significantly correlated with each other: the Pearson r statistic was 0.50 (p = 0.001).

Table 2 presents the Descriptive statistics of the predictor measures.

**Table 2:** Descriptive statistics of the predictor measures.

Theoretical framework	Constructs	N	Alpha	Mean	SD
Theory of Planned Behaviour (TPB)	Attitude direct	2	0.57	5.64	0.99
	Attitude indirect	7	0.76	29.94	6.62
	Subjective Norm	3	0.70	14.88	7.22
	
	Intention	3	0.79	4.90	1.24
	PBC direct	5	0.61	4.53	0.96
	PBC power	10	0.80	3.98	0.97

Social Cognitive Theory (SCT)	Risk perception	6	0.60	4.84	0.79
	Outcome expectancies	9	0.80	24.93	4.68
	Self efficacy	10	0.82	4.55	0.89
	Generalised self efficacy	10	0.87	3.05	0.38

Implementation Intention (II)	Action Planning	-	-	5.15	1.59

Operant Learning Theory (OLT)	Anticipated consequences	3	0.42	4.84	0.89
	Evidence of habitual behaviour	3	0.86	4.37	1.61
	Experienced consequences	4	0.25	0.37	0.86

Common Sense Self regulation Model (CS-SRM)	Identity of condition	2	0.38	3.64	1.26
	Timeline acute	2	0.46	5.50	1.12
	Timeline cyclical	2	0.42	3.49	1.35
	Control (by treatment)	3	0.46	5.89	0.92
	Control (by patient)	3	0.61	5.60	1.11
	Control (by doctor)	2	0.13	5.47	1.00
	Cause a (past care)	1	-	2.67	1.49
	Cause b (exposure to fluoride)	1	-	4.68	1.71
	Cause c (chance or bad luck)	1	-	2.39	1.48
	Cause d (Diet)	1	-	6.59	0.82
	Cause e (oral hygiene)	1	-	6.28	1.21
	Consequence	2	0.411	4.93	1.22
	Emotional Response	4	0.652	3.58	1.11
	Coherence	2	0.524	5.76	1.01

Stage Model	Behavioural Stage*				

Other	Knowledge	7	0.00	3.30	1.10
	
	Behavioural simulation	5	0.68	2.03	1.54

* Stages were distributed as follows: Unmotivated 73 (61%), Motivated (to do more sealants) (13%) Motivated (to do less sealants) 0 (0%); Action (had already something about increasing the number of fissure sealants placed) 31 (26%), Action (had already something about decreasing the number of fissure sealants placed) 1 (1%). Unmotivated 73 (61%) motivated/more sealants (13%) action/more sealants 31 (26%), action/less sealants 1 (1%)

Note: Table 2 reports a description of the constructs as they are used in all the analyses *i.e.*, the final number of items and the final reliabilities, means and SDs.

### Predicting behavioural simulation

In response to the six clinical scenarios, the respondents indicated that they would place PFS for a mean (SD) of 2.03 (1.54) cases.

From Table 3, the constructs that predicted behavioural simulation (*i.e.*, what GDPs said they would do in response to clinical scenarios) were: TPB attitude, subjective norm, perceived behavioural control, and intention; SCT risk perception, outcome expectancies, and self efficacy; II action planning; OLT anticipated consequences, and evidence of habitual behaviour; CS-SRM time (the perception that the onset of caries is acute).

**Table 3:** Predicting behavioural simulation by psychological theory: Correlation and multiple regression analyses.

		Behavioural simulation				
**Theoretical framework**	**Predictive Constructs**	**r**	**Beta**	**R2(adj)**	**df**	**F**

Theory of Planned Behaviour (TPB)^1^	Attitude direct	0.35***				
	Attitude indirect	0.47***	0.29**			
	Subjective Norm	0.18*	0.13			
	PBC direct	0.14				
	PBC power	0.22**	0.05			
	Intention	0.50***	0.32**			
	
				0.31	4, 114	13.99***
	
	PBC power		0.08			
	Intention		0.48***			
	
				0.25	2, 117	20.53***

Social Cognitive Theory (SCT)	Risk perception	0.47***	0.27**			
	Outcome expectancies	0.49***	0.30**			
	Self efficacy	0.29**	0.06			
	Generalised self efficacy	0.06				
	
				0.28	3, 116	16.05***

Implementation intention (II)	Action Planning	0.28***	0.28***	0.07	1, 115	9.84**
Operant Learning Theory (OLT)	Anticipated consequences	0.42***	0.31***			
	Evidence of habitual behaviour	0.49***	0.39***			
	
	Experienced consequences	0.13	0.08			
	
				0.30	3, 115	17.50***

Common Sense Self regulation Model (CS-SRM)	Identity of condition	0.13	0.09			
	Timeline acute	0.22**	0.17			
	Timeline cyclical	-0.08	-0.13			
	Control (treatment)	0.04	0.01			
	Control (patient)	-0.03	-0.06			
	Control (doctor)	0.03	0.02			
	Cause a	-0.14	-0.12			
	Cause b	-0.16	-0.15			
	Cause c	0.12	0.17			
	Cause d	0.00	0.01			
	Cause e	0.00	0.01			
	Consequence	0.14	0.11			
	Emotional Response	-0.00	0.02			
	Coherence	0.03	-0.05			
	
				0.02	14, 97	1.2

Other	Knowledge	-0.06	-0.06	0.00	1, 118	0.4

*p ≤ 0.05; **p ≤ 0.01; ***p ≤ 0.001; r = Pearson product moment correlation coefficient; Beta = standardised regression coefficients. ^1 ^The two blocks in the TPB reflect the two different regression analyses that were run to predict behavioural simulation, one with all the theoretical constructs from the model, and one with only the proximal predictors of behaviour (Intention, PBC). Both direct and indirect measures of PBC and attitude (TPB) were included in this study as each have been used to measure these constructs in the literature. However, only the indirect, theoretically derived measures were included in these theoretical regression equations Similarly, Generalised Self Efficacy was included in this study because this is how some studies using SCT have interpreted and operationalised SE, however only the theoretical measure of SE is included in this theoretical regression equation.

The results of the theory level analyses are shown in Table 3. The TPB explained 31% of the variance in behavioural simulation, SCT explained 29%, II explained 7%, and OLT explained 30%. CS-SRM did not explain significant variance in decision making in the scenarios. The ANOVA for the Stage Model showed that stage did not significantly influence the decision to place a PFS in the behavioural scenarios (F(3,116) = 0.90, p = 0.44).

The theory level analysis for the TPB included only the theoretically derived, indirect measures of Perceived Behavioural Control (PBC) and attitude. However, since these constructs are sometimes operationalised using 'direct' measures, we also included these as alternative measures in this study. Both indirect and direct measures were significantly related to each other (PBC Pearson correlation = 0.36, p < 0.001; attitude Pearson correlation = 0.52, p < 0.001). When direct measures replaced the indirect measures in the theory level regression equation, the TPB explained slightly less variance (F (4,114) = 10.84, p < 0.001; adjusted R^2 ^= 0.25).

In the exploratory cross theory analysis (which included all predictive measures, direct, indirect, general, or specific), habit (OLT), outcome expectancy (SCT), CS-SRM time (acute) were retained in the regression model, together explaining 38% of the variance in the scenario score (Table 4).

**Table 4:** Results of the stepwise regression analyses that included all constructs which significantly predicted outcomes.

Outcome: Behavioural Simulation		Beta	**Adj. R**^2^	df	F
TPB: Attitude Indirect & Direct; Subjective Norm; PBC Power; Intention; SCT: Risk Perception; Outcome expectancy; Self Efficacy; II: Action Planning; CS-SRM: Timeline acute; OLT: anticipated consequences; habit	Habit	0.35			
	
	Outcome expectancy	0.35			
	
	Timeline acute	0.16			
	
			0.38	3, 114	24.6***

**Outcome: Behavioural Intention**					

TPB: Attitude Indirect & Direct; Subjective Norm; PBC Power & PBC Power direct; SCT: Risk Perception; Outcome expectancy Self Efficacy; OLT: anticipated consequences; habit	Habit	0.59			
	
	Attitude Direct	0.25			
	
	Attitude Indirect	0.18			
	
			0.68	3,112	82.5***

***p < 0.001; Beta = standardised regression coefficient; TPB = Theory of Planned Behaviour; PBC = perceived behavioural control; SCT = Social Cognitive Theory; CS-SRM = Common Sense Self-Regulation Model; II - Implementation Intention; OLT = Operant Learning Theory

### Predicting behavioural intention

The mean (SD) for intention was 4.90 (1.24) from a possible score of 7 (strongest intention to place a PFS. The constructs that predicted behavioural intention were: TPB attitude, perceived behavioural control; SCT risk perception, outcome expectancies; OLT anticipated consequences, and evidence of habitual behaviour (Table 5).

**Table 5:** Predicting behavioural intention by psychological theory: Correlation and multiple regression analyses.

		Behavioural intention				
**Theoretical framework**	**Predictive Constructs**	**r**	**Beta**	**R2(adj)**	**df**	**F**

Theory of Planned Behaviour (TPB)^1^	Attitude direct	0.54***				
	Attitude indirect	0.52***	0.47***			
	Subjective Norm	0.17	0.18*			
	PBC direct	0.22**				
	PBC power	0.28*	0.15			
	Intention					
	
				0.30	3, 115	17.83***

Social Cognitive Theory (SCT)	Risk perception	0.42***	0.19*			
	Outcome expectancies	0.49*	0.39***			
	Self efficacy	0.21	0.04			
	Generalised self efficacy	0.09				
	
				0.25	3, 116	14.21***

Operant Learning Theory (OLT)	Anticipated consequences	0.42***	0.20***			
	Evidence of habitual behaviour	0.75***	0.69***			
	
	Experienced consequences	0.17	0.06			
	
				0.58	3, 115	55.40***

Common Sense Self regulation odel (CS-SRM)	Identity of condition	0.05	0.03			
	Timeline acute	0.08	0.03			
	Timeline cyclical	-0.14	-0.16			
	Control (treatment)	-0.01	-0.11			
	Control (patient)	0.03	0.05			
	Control (doctor)	0.10	0.13			
	Cause a	-0.05	-0.07			
	Cause b	-0.15	-0.16			
	Cause c	0.05	0.11			
	Cause d	-0.03	-0.07			
	Cause e	0.12	0.21			
	Consequence	0.15	0.10			
	Emotional Response	0.13	0.09			
	Coherence	-0.03	-0.06			
	
				0.01	14, 97	1.10

Other	Knowledge	0.02	0.02	0.00	1,118	0.30

*p ≤ 0.05; **p ≤ 0.01; ***p ≤ 0.001; r = Pearson product moment correlation coefficient; Beta = standardised regression coefficients. ^1 ^Both direct and indirect measures of PBC and attitude (TPB) were included in this study as each have been used to measure these constructs in the literature. However, only the indirect, theoretically derived measures were included in these theoretical regression equations Similarly, Generalised Self Efficacy was included in this study because this is how some studies using SCT have interpreted and operationalised SE, however only the theoretical measure of SE is included in this theoretical regression equation. II is a post intention theory and so is not included in this analysis.

The results of the theory level analyses are also shown in Table 4. The TPB explained 30% of the variance in behavioural intention, SCT explained 16%, OLT explained 57%, CS-SRM explained 1%, and knowledge explained 0%.

When direct measures replaced the indirect measures in the TPB theory level regression equation, the results were essentially unchanged (F (3,115) = 17.84, p < 0.001; adjusted R^2 ^= 0.30).

The ANOVA for the stage model showed that stage did significantly predict intention to place a PFS (F(3, 119) = 5.66, p = 0.001). Post hoc comparison of means indicated that the dentists in the action stage (had already something about increasing the number of PFS placed) had significantly higher intention of placing PFS than dentists in the unmotivated or motivated stages.

In the cross theory analysis, only OLT evidence of habitual behaviour and TPB attitudes were retained in the regression model, together explaining 68% of the variance in intention (Table 4).

## Discussion

The objective of this study was to identify factors derived from psychological models predictive of an evidence-based clinical behaviour, the placing of PFS in six to sixteen year old patients in Scotland. A theory-based questionnaire was developed to assess constructs from six models and applied to the prediction of clinical decision-making based on scenarios (behavioural simulation), as well as dentists' intention to place PFS to manage caries in this age group.

Of the six models, only the CS-SRM did not explain a significant proportion of the variance in both behavioural simulation and intention. Only behavioural stage did not account for significant variance in behavioural simulation. The usual approaches to measuring behavioural stage in the literature were used in this study, but a more complex approach may be more informative in terms of the number and the nature of the stages when applied to clinical decision-making in specific situations (as depicted by the scenarios) rather than to a general intention.

Why the CS-SRM does not appear to be working is also open to discussion, because both theoretical and measurement explanations are possible. The internal reliability of the measures for this theory was consistently poor. The measures in this study were derived from a standardized measure developed for the point of view of the patient, and it may be that the items were not adequately adapted for the point of view of the clinician. Theoretically, representations of someone else's 'illness' may not influence the individual dentist's 'self-regulation'. It is also possible that illness representations *per se *simply do not drive clinical behaviour, that is, dentists' perceptions about caries as a disease in and of itself does not influence their decision to place PFS. This interpretation was supported by anecdotal evidence during the preliminary study interviews, as well as similar results from surveys using this model to predict other clinical behaviours [21,22]. However, more work is required to address the issue of whether the lack of predictive power for this model is either measure-, theory-, or behaviour-related.

Nevertheless, the constructs within all models acted in line with theoretical predictions. The likelihood of a decision in favour of fissure sealing increased with stronger intention to do so, more positive attitude, greater perceived behavioural control, greater self-efficacy, higher risk perceptions, more positive outcome expectancies, experience of reinforcing consequences, if dentists had a prior action plan about placing PFS, and if placing PFS was perceived as habitual. Also, dentists in the action stage had significantly higher intention of placing PFS than dentists in the unmotivated or motivated stages. This is a correlational study, so the causative aspects of the theories and constructs remain untested in this population; but it is promising for the utility of applying psychological theory to changing clinical behaviour that the constructs are acting as the theories expect. These results suggest that an intervention that specifically targets predictive factors may have the greatest likelihood of success in influencing the implementation of this evidence-based practice.

To further refine possible intervention targets and their operationalisation, an aggregated, cross theory analysis was performed, which included all predictive measures used in this study. This stepwise regression analyses revealed that the main constructs driving GDPs' decision to place PFS in specific scenario situations was habit, with additional influence from outcome expectancies, and the belief that caries was a condition with an acute onset. The main constructs driving GDPs' general intention to place PFS was habit, with additional influence from both operationalisations of attitude (direct and indirect). Taken together, the results suggest that participating dentists operate in a predominantly habitual manner backed up by beliefs that support their habit. This is anecdotally supported by the preliminary study of independent GDPs, when dentists tended to fall into two camps -- those who claimed they always included the placement of PFS (both preventive and restorative) in their usual management of child patients, and dentists who rarely or never included fissure sealing in their child patient management repertoire. That our measure of habit was the only variable to consistently predict both outcome measures provides support of this being a general phenomenon. This suggests that influencing this clinical behaviour may require an intervention targeted at helping dentists change their beliefs about the consequences of placing PFS, and one that enables them to habitually incorporate PFS as part of their usual routine when dealing with managing caries in children.

The cross-theory stepwise regression models (Table 4) explained more variance in both outcomes than any of the theoretical models included. This may indicate that clinical behaviour requires a more sophisticated explanatory model than those used here, one that incorporates motivational and action elements. Similar results were found in the study examining the relationship between these models and taking dental radiographs [21]. Additionally, when used with different clinical groups (GPs, dentists), different constructs predicted different proportions of the variance in intention and behaviour relating to placing PFS, taking radiographs, and managing upper respiratory tract infections without antibiotics [22].

Our inclusion of multiple models all directed at understanding behaviour, meant that this study included a number of similar or overlapping constructs (*e.g.*, PBC and self-efficacy), and direct and indirect operationalisations of the same construct (*e.g.*, attitude and PBC). We also included only the theoretically determined (indirect) measures in the theory-specific regressions (Table 3 and Table 5), however both direct and indirect measures of attitude together accounted for significant variance in behavioural intention in the stepwise analysis (Table 4), with no evidence of co-linearity problems. The predictive success of the majority of these models and the implications of the results of the stepwise analyses, raise the question of what would be an optimum core set of theories and measures if the aim was to develop a framework to cover most clinical behaviours and clinical groups to apply in implementation research. A more complex framework incorporating both reflective reasoning (about the consequences of action) and less reflective associative or habitual processes may be needed to describe the processes involved in clinical decision-making, as for example, that described by Strack and Deutsch [38]. Michie *et al. *[39] have also developed a framework, identifying 12 theoretical domains collating a number of similar constructs and measures, that could be considered in research into understanding and changing behaviour. The current study investigated several of these theoretical domains, and at least three domains had significant coefficients in the stepwise regression model. Future research needs to further explore whether theories, constructs, and the operationalisation of constructs, can be consistently predictive across a range of clinical behaviours before a final rationale can be developed for choosing theory, theoretical components, and their operationalisations to apply in implementation research.

Because encouraging the implementation of any evidence-based practice commonly entails various methods of increasing knowledge, knowledge was also included as a predictive construct in this study. The knowledge measure included questions about both how and why PFS might be used in the management of caries, both as preventive and restorative treatment. However, knowledge was not related to either outcome variable. It is possible that this result may be due to the poor internal consistency of this measure, which covered both specific and general issues relating to the targeted clinical behaviour. Nevertheless, this result suggests that implementation interventions that specifically target knowledge may not influence this behaviour. This is supported by the results of another study that investigated the effect of an educational intervention on placing PFS [40]. They indeed found that an educational strategy had no effect on the number of PFS placed, despite high uptake of the education offered, suggesting that behaviour change strategies aimed at changing knowledge alone are unlikely to be successful in this clinical area, and adding to the evidence that increasing knowledge is generally not enough to change clinical practice.

It was not possible to explicitly assess a behavioural outcome because PFS in Scotland came under a total capitation fee. In future studies of this kind, it will be important to invest more in the measurement of behavioural data, particularly when not routinely collected. Nevertheless, the outcomes used to proxy this clinical behaviour were to some extent validated by the results of an independent study in which participating dentists were paid to specifically keep records of the number of PFS they placed [41]. Although dealing with a more limited patient population and management strategy (placing PFS only on second molars in 11 to 14 year olds) than here (placing PFS on any teeth in six to sixteen year old patients), actual clinical behaviour was significantly predicted by the same models which predicted the proxy outcomes in this study.

Operationalising the constructs with theoretical purity was a challenge. For example, the preliminary study revealed that it was difficult to ask dentists about their control over placing PFS because they believed that, even if they felt there were barriers to performing the behaviour, ultimately they had total control because only they decided if the behaviour was to be performed. At the theoretical level, a number of the models (OLT, II, CS-SRM) have not previously been operationalised in this way, except in our parallel studies. In particular, OLT and II are more usually used as intervention methods to change behaviour. This meant that we had to both define and develop measures of their 'active ingredients' to serve as predictive components. Although we did this by literature review and expert forum (see below), it may be argued that these derived components may lack validity. However, the measures of each of the theoretical constructs adhered as closely as possible to any operational instruction from the theory creator(s), when it existed. Every item making up each construct was also discussed in a forum of experts, including three psychologists with experience of operationalising these models, until a consensus was reached, providing face and content validity of the measures as much as possible. Further evidence that the models were successfully operationalised was provided by the constructs' performance being in line with theoretical expectations. Also, the variance explained in behavioural simulation and behavioural intention was slightly better than expected from systematic reviews including many of these constructs [15,18]. Indeed, a major strength of this study is the qualitative preparatory research that went into the design of the questionnaire and the operationalisation of the theoretical models.

Our final response rate was not high compared to what would be expected for a postal questionnaire in medicine (approximately 60%) [42,43]. This may mean that our participants were not a representative sample. Nevertheless, our respondents appear well-matched with the overall population of GDPs in Scotland, as well as participants in our previous study using a similar questionnaire to investigate the taking of dental radiographs in Scotland [21], which achieved a response rate of 40%, and the Randomized controlled trial of GDPs in Scotland, which achieved a response rate of 47% [40]. Furthermore, if our participants were restricted to a sample of keen, evidence-compliant dentists, then they should have decided to place PFS in all five scenario situations (the evidence-based outcome). However, on average, participating dentists decided that placing PFS would be appropriate in only two scenarios -- and rarely the same two scenarios. This appears to reflect the current poor behaviour of GDPs in Scotland, further supporting the lack of bias in this sample. The relatively poor response rate also meant that regression analyses that included many predictors were underpowered. This may account for the lack of success of the CS-SRM, although it is difficult to determine how power, measurement or theoretical issues contribute individually or in combination to this problem.

The overall results of this study are similar to other studies applying a range of theoretical models to clinical decision-making [21,22,40] and it is possible that this may reflect the nature of the behaviours examined. Both the placing of PFS and taking intra-oral radiographs are desired behaviours that are not currently fully implemented by dentists. Further work is required to explore whether psychological theories, as well as variables derived from these theories, can be consistently predictive across a range of clinical behaviours before a rationale can be developed for choosing theory or theoretical components to apply in implementation research.

## Summary

This study provides further evidence that psychological models can be useful in understanding and predicting clinical behaviour. The focus on multiple psychological theories provides depth and focus that may be generalisable across different behaviours as well as different populations, and takes advantage of decades of research specifically into the antecedents of behaviour and methods of behaviour change. To encourage the particular behaviour of placing PFS, the data suggest that interventions should be directed at changing habits and beliefs about the outcomes of this behaviour. However, there remain conceptual and methodological challenges when operationalising some psychological models, particularly in the same instrument, that need to be overcome when using this method of understanding and predicting clinical behaviour in future.

## Competing interests

The authors declare that they have no competing interests.

## Authors' contributions

AW, MPE, JMG, MJ, and NP conceived the study. DB, JC, NP, and LG contributed to the daily running of the study. DB, JC, and NP oversaw the analysis that was conducted by GM. All authors commented on sequential drafts of the paper and agreed the final draft.

## Supplementary Material

Additional file 1 **Measuring a behavioural proxy for the placing a preventative fissure sealant (PFS)**. Additional text relating to measurement of a behavioural proxy for the placing a preventative fissure sealant (PFS). Click here for file

Additional file 2 **Questionnaire Index**. Index to the PRIME Fissure Sealant Questionnaire. Click here for file

Additional file 3 **Questionnaire**. Clinical practice survey: the use of fissure sealants in managing 6 to 16 year old patients. Click here for file
