# Supplementary material for: Applying psychological theories to evidence-based clinical practice: identifying factors predictive of placing preventive fissure sealants
Source: Implement Sci. 2010 Apr 8;5:25. doi: 10.1186/1748-5908-5-25 (PMC2864198; doi:10.1186/1748-5908-5-25)
Supplement: Additional file 1 — Measuring a behavioural proxy for the placing a preventative fissure sealant (PFS). Additional text relating to measurement of a behavioural proxy for the placing a preventative fissure sealant (PFS). [file 1748-5908-5-25-S1.DOC]

**Additional File 1: Measuring a behavioural proxy for the placing a preventative fissure sealant (PFS)**

Although it was not possible to measure PFS, the study team had intended to include a clinical behaviour to proxy placing PFS as well as the simulation and intention measures. Although not commonly employed as a restorative treatment, a fissure sealant can be used in place of an amalgam filling when caries is present. Like amalgam fillings, there is an itemised fee for service for restorative fissure sealants, which means that there was data available for the number of restorative fissure sealants (RFS) placed by each dentist in Scotland. The study team reasoned that dentists who tended to place less PFS – a behaviour supported by the best evidence available - should be even less likely to use fissure sealants restoratively, where little evidence exists supporting this behaviour. However, so few were placed by each dentist (mean = 0.04, SD =0.06) over the relevant 12 month period (6 months prior and 6 months after the questionnaire posting) that there was not enough behavioural data to allow discriminating matching to the predictor variables. No further analysis of behavioural data was therefore performed.
