# Supplementary material for: Applying psychological theories to evidence-based clinical practice: identifying factors predictive of placing preventive fissure sealants
Source: Implement Sci. 2010 Apr 8;5:25. doi: 10.1186/1748-5908-5-25 (PMC2864198; doi:10.1186/1748-5908-5-25)
Supplement: Additional file 2 — Questionnaire Index. Index to the PRIME Fissure Sealant Questionnaire. [file 1748-5908-5-25-S2.DOC]

**Additional file 2**

Index to the PRIME Fissure Sealant Questionnaire

| **Section 1** | ***Construct*** | ***Theory*** |
| --- | --- | --- |
| 1a-c | Subjective Norm (Normative Beliefs) | TPB |
| 2 | Self Expectancy | SCT |
| 3 | Self Expectancy | SCT |
| 4a-b | Risk perception | SCT |
| 5a-h | Attitude (Outcome expectancies) | TPB |
| 6,7 | Past behaviour | - |
| 8a-b | Intention | TPB |
| 9a-b | Anticipated Consequences | SCT |
| 10,11,12,13 | Evidence of Habit | OpC |
| 14 | Intention | TPB |
| 15 | preventive program attitude | - |
| 16-17 | Prior Planning | ImpI |
| 18a-d | Experienced Consequences | OpC |
| 19 | Stage | Stage |
| 20a-g | Perceived Behavioural Control (Control beliefs) | TPB |
| 21a-c | Perceived Behavioural Control (Control beliefs) | TPB |
| 22a-e | Perceived Behavioural Control (General) | TPB |
| 23a-b | Attitude (General) | TPB |
| 24,25 | Intention | TPB |
| 26a-c | Subjective Norm (Motivation to comply) | TPB |
| 27a-h | Attitude (Outcome evaluations) | TPB |
| 27i-j | Self evaluations | SCT |
| 28a-c | Self-efficacy | SCT |
| 29a-g | Self-efficacy | SCT |
| 30 | Prior Planning | ImpI |
| **Section 2** | | |
| 1a-c | Identity | SR |
| 1d-f | Time | SR |
| 2a-e | Cause | SR |
| 3 | Time | SR |
| 4-6 | Control - treatment | SR |
| 7-8 | Control - patient | SR |
| 9-10 | Control - dentist | SR |
| 11-13 | Consequence | SR |
| 14-15 | Coherence | SR |
| 16-19 | Emotion | SR |
| 16-19 | Emotion | SR |
| 20a-b | Knowledge | KAB |
| **Section 3** | | |
| 1a-e, 2 | Knowledge | KAB |
| **Section 4** | | |
| 1-6 | Behavioural Simulation (scenarios) | - |
