# Supplementary material for: Applying psychological theories to evidence-based clinical practice: identifying factors predictive of placing preventive fissure sealants
Source: Implement Sci. 2010 Apr 8;5:25. doi: 10.1186/1748-5908-5-25 (PMC2864198; doi:10.1186/1748-5908-5-25)
Supplement: Additional file 3 — Questionnaire. Clinical practice survey: the use of fissure sealants in managing 6 to 16 year old patients. [file 1748-5908-5-25-S3.DOC]

**Clinical Practice Survey:**

**The use of Fissure Sealants in managing 6 to 16 year old patients**

office use only

First of all, thank you for participating in this research. This questionnaire contains items about elements that may influence your decision to use fissure sealants in the management of 6 to 16 year old patients. The questions are based on a previous study of GDP attitudes toward fissure sealants. You will notice that some of the questions are very alike – this is **not** meant to catch any one out. Sometimes questions posed in slightly different ways can mean different things to people, and this is something we are trying to understand - so please answer all the items without making a deliberate effort to be consistent. **This is not a test.** Dentists work in a wide range of contexts and treat patients from a variety of backgrounds, so we expect everyone to have different ideas and experiences, which will be reflected in their answers. The main thing to remember is to answer what first comes to mind, not what you believe you should answer or what we want to see – this is the only way progress can be made in understanding the management of patients in the ‘real world’. Your responses will be held in confidence.

#### Participation Slip

|  |  | Please mark the appropriate box | | | | |
| --- | --- | --- | --- | --- | --- | --- |
| Yes, I would like to participate in this study. I understand this means completing the questionnaire and giving permission for the PRIME research team to obtain data relating to restorations and practice profiles from MIDAS *(please note that we need you to mark this box even if you have completed the questionnaire so that we can fully include your views in this study)* | |  | | 1. | |  |
| *We appreciate that some participants would like to be informed about the end-result of projects that they are involved in. Please tick here if you would like to receive a summary of the results.* | | |  | |  | |

| No, I do not wish to participate in this study | 2. |
| --- | --- |

Signature…………………………………………… Date….../….../……...

Please check that your contact details are correct and amend if necessary.

| **Your Contact Phone Number** |  | **e-mail** |  |
| --- | --- | --- | --- |

# *This page will be removed and stored separately from the remainder of the questionnaire to preserve confidentiality*

# Background

| **Male**  For office use only |  | **Female** |  |
| --- | --- | --- | --- |

| 1. How long have you been qualified as a dentist? |  |  |  | **Years** |
| --- | --- | --- | --- | --- |

| 2 How many dentists (excluding you) are there in your practice? |  |  |  |
| --- | --- | --- | --- |

| 3. Are you now, or within the past 2 years been, a vocational dental trainer? | Yes |  | No |  |  |
| --- | --- | --- | --- | --- | --- |

| 4. How many clinical sessions (0.5 days) do you work per week on average? |  |  |  |
| --- | --- | --- | --- |

| 5. Does your practice employ dental hygienists? | Yes |  | No |  |  |
| --- | --- | --- | --- | --- | --- |

| 6. What is your ***approximate***  total **practice** list size? |  |  |  |  |  |  |  |
| --- | --- | --- | --- | --- | --- | --- | --- |

| 7. ***Approximately*** what proportion of your 6 to 16 year old patients do you consider to have a Moderate to High risk of caries? |  | **%** | **Not sure** |  |  |
| --- | --- | --- | --- | --- | --- |

**Section 1: placing Fissure Sealants**

***Please treat all sub-questions, e.g. 1a) b) c), as separate questions and answer each one.***

***1. I feel under pressure:***  Strongly Disagree Strongly Agree

| a) From patients’ parents to place fissure sealants…………………… | **1** | **2** | **3** | **4** | **5** | **6** | **7** |
| --- | --- | --- | --- | --- | --- | --- | --- |
| b) From Post Graduate courses to place fissure sealants …………… | **1** | **2** | **3** | **4** | **5** | **6** | **7** |
| c) From the Dental Practice Board to place fissure sealants ……….… | **1** | **2** | **3** | **4** | **5** | **6** | **7** |
| 2. If I place fissure sealants, then I will think of myself as a caring dentist………………………………………………………………….. | **1** | **2** | **3** | **4** | **5** | **6** | **7** |
| 3. If I place fissure sealants, then I will think of myself as a competent dentist……..…...………..……..……………………….… | **1** | **2** | **3** | **4** | **5** | **6** | **7** |
| 4a) It is highly likely that children with ***medium to high*** risk of caries will be worse off if I do not place fissure sealants………………… | **1** | **2** | **3** | **4** | **5** | **6** | **7** |
| b) It is highly likely that children with ***low risk*** of caries will be worse off if I do place fissure sealants…...………..……………..... | **1** | **2** | **3** | **4** | **5** | **6** | **7** |

# 5. In general, placing fissure sealants: Strongly Disagree Strongly Agree

| a) Effectively reduces caries risk ………………………………………. | **1** | **2** | **3** | **4** | **5** | **6** | **7** |
| --- | --- | --- | --- | --- | --- | --- | --- |
| b) Is a good way to acclimatize children to dentistry………………. | **1** | **2** | **3** | **4** | **5** | **6** | **7** |
| c) Reduces the need for more radical treatment in the future……. | **1** | **2** | **3** | **4** | **5** | **6** | **7** |
| d) Encourages future attendance ……………………………………. | **1** | **2** | **3** | **4** | **5** | **6** | **7** |
| e) Allows me to feel personal/professional satisfaction…………… | **1** | **2** | **3** | **4** | **5** | **6** | **7** |
| f) Makes future problems more difficult to detect………………….. | **1** | **2** | **3** | **4** | **5** | **6** | **7** |
| g) Disadvantages me (e.g. financially, time)………………………… | **1** | **2** | **3** | **4** | **5** | **6** | **7** |

6. From memory, how many of the ***last ten*** of your 6 to 16 yr old patients were given at least one fissure sealant?

| **0** | **1** | **2** | **3** | **4** | **5** | **6** | **7** | **8** | **9** | **10** |
| --- | --- | --- | --- | --- | --- | --- | --- | --- | --- | --- |

7. From memory, ***approximately*** what proportion of children ***last month*** were given a fissure sealant? **_________%**

8a) For ***every ten*** children you see, how many would you ***expect*** to get at least one fissure sealant?

| **0** | **1** | **2** | **3** | **4** | **5** | **6** | **7** | **8** | **9** | **10** |
| --- | --- | --- | --- | --- | --- | --- | --- | --- | --- | --- |

b) ***Approximately*** what proportion of children you see ***next month*** would you ***expect***  to get a fissure sealant?.**______%**

### 9. If I routinely place fissure sealants: Strongly Disagree Strongly Agree

| a) On balance, my life will be easier in the long run………………… | **1** | **2** | **3** | **4** | **5** | **6** | **7** |
| --- | --- | --- | --- | --- | --- | --- | --- |
| b) On balance, the consequences (e.g. stress, time, future treatment requirements) will make me generally worse off………………….. | **1** | **2** | **3** | **4** | **5** | **6** | **7** |
| 10. It is highly likely that my 6 to 16 year old patients will get caries a) If I ***do*** fissure seal………………………………………..………… | **1** | **2** | **3** | **4** | **5** | **6** | **7** |
| b) If I ***do not*** fissure seal………………………………………..……. | **1** | **2** | **3** | **4** | **5** | **6** | **7** |
| 11. When I see a 6 to 16 year old patient, I automatically consider placing a fissure sealant……………………………………………… | **1** | **2** | **3** | **4** | **5** | **6** | **7** |
| 12. It is my usual practice to place fissure sealants on a) erupting 6s | **1** | **2** | **3** | **4** | **5** | **6** | **7** |
| b) erupting 7s | **1** | **2** | **3** | **4** | **5** | **6** | **7** |
| 13. I aim to place fissure sealants as part of 6 to 16 year old patient management ………………………………….……………….……... | **1** | **2** | **3** | **4** | **5** | **6** | **7** |
| 14. The last time I placed a fissure sealant, the overall result, from my point of view, was good…………………………………….………. | **1** | **2** | **3** | **4** | **5** | **6** | **7** |
| 15. A preventive program aimed at controlling small lesions at the enamel stage would not be as successful as conventional restorations in managing caries of children ***in my practice***……… | **1** | **2** | **3** | **4** | **5** | **6** | **7** |
| 16. Currently, my standard method of managing caries does not primarily include placing fissure sealants……………………………. | **1** | **2** | **3** | **4** | **5** | **6** | **7** |

17a) Think about the last time you decided to place a fissure sealant in a 6 to 16 year old patient and **felt pleased** that you had done so. Do you think the result of this episode has made you:

|  | More likely  to place a  fissure sealant |  | **Less likely**  **to place a fissure sealant** |  | **Unchanged** |  | **Not sure** |  | **Never occurred** |  |
| --- | --- | --- | --- | --- | --- | --- | --- | --- | --- | --- |

b) Think about the last time you decided to place a fissure sealant in a 6 to 16 year old patient and **felt sorry** that you had done so. Do you think the result of this episode has made you:

|  | More likely  to place a  fissure sealant |  | **Less likely**  **to place a fissure sealant** |  | **Unchanged** |  | **Not sure** |  | **Never occurred** |  |
| --- | --- | --- | --- | --- | --- | --- | --- | --- | --- | --- |

c) Think about the last time you decided **not to** place a fissure sealant in a 6 to 16 year old patient and **felt pleased** that you had not done so. Do you think the result of this episode has made you:

|  | More likely  to place a  fissure sealant |  | **Less likely**  **to place a fissure sealant** |  | **Unchanged** |  | **Not sure** |  | **Never occurred** |  |
| --- | --- | --- | --- | --- | --- | --- | --- | --- | --- | --- |

d) Think about the last time you decided **not to** place a fissure sealant in a 6 to 16 year old patient and **felt sorry** that you had not done so. Do you think the result of this episode has made you:

|  | More likely  to place a  fissure sealant |  | **Less likely**  **to place a fissure sealant** |  | **Unchanged** |  | **Not sure** |  | **Never occurred** |  |
| --- | --- | --- | --- | --- | --- | --- | --- | --- | --- | --- |

18. Which of these sentences *most* characterises you at the moment:

Please tick *only one* of the 7 boxes)

| **1.** | I have not yet thought about changing the number of fissure sealants that I currently do |
| --- | --- |
| **2.** | It has been a while since I have thought about changing the number of fissure sealants that I do |
| **3.** | I have thought about it and decided that I *will not change* the number of fissure sealants I do |
| **4** | I have decided that I *will do more* fissure sealants |
| **5.** | I have decided that I *will do less* fissure sealants |
| **6.** | I have *already* done something about increasing the number of fissure sealants I do |
| **7.** | I have *already* done something about decreasing the number of fissure sealants I do |

### 19. I find it difficult to decide in favour of placing a fissure sealant if: Strongly Disagree Strongly Agree

| a) The patient is a poor attender………………………………………… | **1** | **2** | **3** | **4** | **5** | **6** | **7** |
| --- | --- | --- | --- | --- | --- | --- | --- |
| b) The 7s are erupting and the 6s are not sealed……………………… | **1** | **2** | **3** | **4** | **5** | **6** | **7** |
| c) The child is disinterested/not responsive to advice……………… | **1** | **2** | **3** | **4** | **5** | **6** | **7** |
| d) The child’s oral hygiene is excellent………………………………… | **1** | **2** | **3** | **4** | **5** | **6** | **7** |
| e) The child is older (both 6s and 7s erupted)………………………… | **1** | **2** | **3** | **4** | **5** | **6** | **7** |
| f) The parent is unmotivated when it comes their child’s teeth……. | **1** | **2** | **3** | **4** | **5** | **6** | **7** |
| g) There is a lot of decay in the mouth………………………………… | **1** | **2** | **3** | **4** | **5** | **6** | **7** |

### 20. I find it difficult to effectively place a fissure sealant if: Strongly Disagree Strongly Agree

| a) The child has poor oral hygiene | **1** | **2** | **3** | **4** | **5** | **6** | **7** |
| --- | --- | --- | --- | --- | --- | --- | --- |
| b) There is poor moisture control | **1** | **2** | **3** | **4** | **5** | **6** | **7** |
| c) The mouth is small | **1** | **2** | **3** | **4** | **5** | **6** | **7** |
| 21 a) I would like to manage caries in children by placing fissure sealants, but I don’t really know if I can…………………………... | **1** | **2** | **3** | **4** | **5** | **6** | **7** |
| b) It is entirely up to me whether I place fissure sealants……………. | **1** | **2** | **3** | **4** | **5** | **6** | **7** |
| c) I am confident that I can place fissure sealants in 6 to 16 yr olds whenever I want ……………………………………………………… | **1** | **2** | **3** | **4** | **5** | **6** | **7** |
| d) I can overcome all obstacles, whatever they may be, in regard to placing fissure sealants ……………………………………………… | **1** | **2** | **3** | **4** | **5** | **6** | **7** |
| e) Generally, I find it easy to place fissure sealants in children…..… | **1** | **2** | **3** | **4** | **5** | **6** | **7** |
| 22 a) In general, the possible harm caused by placing fissure sealants is outweighed by its benefits……………………………………… | **1** | **2** | **3** | **4** | **5** | **6** | **7** |
| b) In general, placing fissure sealants is more often bad practice than good practice………………………………………….……… | **1** | **2** | **3** | **4** | **5** | **6** | **7** |
| 23. I have in mind to place fissure sealants when I see 6 to 16 yr olds.. | **1** | **2** | **3** | **4** | **5** | **6** | **7** |
| 24. I intend to place fissure sealants as a primary part of managing caries in 6 to 16 year old patients……………………………………. | **1** | **2** | **3** | **4** | **5** | **6** | **7** |

### 25. How motivated are you to do: Not at All Very Much

| a) What patients’ parents think you should…………………………. | **1** | **2** | **3** | **4** | **5** | **6** | **7** |
| --- | --- | --- | --- | --- | --- | --- | --- |
| b) What Post Graduate courses say you should…………………….. | **1** | **2** | **3** | **4** | **5** | **6** | **7** |
| c) What the Dental Practice Board says you should………………… | **1** | **2** | **3** | **4** | **5** | **6** | **7** |

***26. In general:*** Important Unimportant

| a) Reducing caries risk is…………………………………………..……. | **1** | **2** | **3** | **4** | **5** | **6** | **7** |
| --- | --- | --- | --- | --- | --- | --- | --- |
| b) Acclimatizing children to dentistry is…………………………..….. | **1** | **2** | **3** | **4** | **5** | **6** | **7** |
| c) Reducing the need for more radical treatment is…………….……. | **1** | **2** | **3** | **4** | **5** | **6** | **7** |
| d) Encouraging future attendance is……………………..……………. | **1** | **2** | **3** | **4** | **5** | **6** | **7** |
| e) Personal/Professional satisfaction is……………………………….. | **1** | **2** | **3** | **4** | **5** | **6** | **7** |
| g) Detecting problems underneath a sealant in future is…………. | **1** | **2** | **3** | **4** | **5** | **6** | **7** |
| h) Not putting yourself at any disadvantage is………………………. | **1** | **2** | **3** | **4** | **5** | **6** | **7** |
| i) Thinking of yourself as a caring dentist is…………………………… | **1** | **2** | **3** | **4** | **5** | **6** | **7** |
| j) Thinking of yourself as a competent dentist is……………………… | **1** | **2** | **3** | **4** | **5** | **6** | **7** |

### 27. How confident are you that you can effectively place a

### fissure sealant in a 6 to 16 yr old if: Not at all Confident Extremely Confident

| a) The child has poor oral hygiene…………………………………….. | **1** | **2** | **3** | **4** | **5** | **6** | **7** |
| --- | --- | --- | --- | --- | --- | --- | --- |
| b) There is poor moisture control……………………………………… | **1** | **2** | **3** | **4** | **5** | **6** | **7** |
| c) The mouth is small……………………………………………………. | **1** | **2** | **3** | **4** | **5** | **6** | **7** |

### 28. How confident are you that you can decide to fissure seal if:

| a) The patient is a poor attender………………………………………… | **1** | **2** | **3** | **4** | **5** | **6** | **7** |
| --- | --- | --- | --- | --- | --- | --- | --- |
| b) The 7s are erupting and the 6s are not sealed……………………… | **1** | **2** | **3** | **4** | **5** | **6** | **7** |
| c) The child is disinterested/not responsive to advice……………… | **1** | **2** | **3** | **4** | **5** | **6** | **7** |
| d) The child’s oral hygiene is excellent………………………………… | **1** | **2** | **3** | **4** | **5** | **6** | **7** |
| e) The child is older (both 6s and 7s erupted)………………………… | **1** | **2** | **3** | **4** | **5** | **6** | **7** |
| f) The parent is unmotivated when it comes their child’s teeth……. | **1** | **2** | **3** | **4** | **5** | **6** | **7** |
| g) There is a lot of decay in the mouth………………………………… | **1** | **2** | **3** | **4** | **5** | **6** | **7** |

### 29a) Do you have a clear idea how you would want to manage caries in 6 to 16 year olds?

### Yes No Unsure

| b) If Yes, Please describe it briefly: |
| --- |
|  |
|  |
|  |

## Section 2: Caries

### 1. Caries is a condition: Strongly Disagree Strongly Agree

| a) With symptoms generally of an intense nature………………. | **1** | **2** | **3** | **4** | **5** | **6** | **7** |
| --- | --- | --- | --- | --- | --- | --- | --- |
| b) With many symptoms ………………………………………...… | **1** | **2** | **3** | **4** | **5** | **6** | **7** |
| c) Which should concern dentists…………………………………. | **1** | **2** | **3** | **4** | **5** | **6** | **7** |

***1. Caries is a condition: Strongly Disagree Strongly Agree***

| d) Which is likely to be permanent rather than temporary…….. | **1** | **2** | **3** | **4** | **5** | **6** | **7** |
| --- | --- | --- | --- | --- | --- | --- | --- |
| e) Which will pass quickly…………………………………………. | **1** | **2** | **3** | **4** | **5** | **6** | **7** |
| f) Which is very unpredictable…………………………………….. | **1** | **2** | **3** | **4** | **5** | **6** | **7** |

**2. Caries is caused by: *Strongly Disagree Strongly Agree***

| a) Poor care from dentists in the past…………………………… | **1** | **2** | **3** | **4** | **5** | **6** | **7** |
| --- | --- | --- | --- | --- | --- | --- | --- |
| b) Low exposure to fluoride ………………………………………. | **1** | **2** | **3** | **4** | **5** | **6** | **7** |
| c) Chance or bad luck………………………………………………. | **1** | **2** | **3** | **4** | **5** | **6** | **7** |
| d) Diet………………………………………………………………… | **1** | **2** | **3** | **4** | **5** | **6** | **7** |
| e) Poor oral hygiene………………………………………………. | **1** | **2** | **3** | **4** | **5** | **6** | **7** |
| 3. The symptoms of caries change a great deal from day to day…….. | **1** | **2** | **3** | **4** | **5** | **6** | **7** |
| 4. There is very little that can be done to arrest the progress of caries. | **1** | **2** | **3** | **4** | **5** | **6** | **7** |
| 5. There is nothing which can prevent caries…………………………... | **1** | **2** | **3** | **4** | **5** | **6** | **7** |
| 6. Treatment can control caries………………………………………….. | **1** | **2** | **3** | **4** | **5** | **6** | **7** |
| 7. There is a lot which the patient can do to control the symptoms or signs of caries………………………………………………………….. | **1** | **2** | **3** | **4** | **5** | **6** | **7** |
| 8. What the patient does can determine whether a) caries reverses | **1** | **2** | **3** | **4** | **5** | **6** | **7** |
| b) caries progresses | **1** | **2** | **3** | **4** | **5** | **6** | **7** |
| 9. Nothing I do will affect the progression of caries…………………... | **1** | **2** | **3** | **4** | **5** | **6** | **7** |
| 10. What I do can determine whether the patient’s caries reverses….. | **1** | **2** | **3** | **4** | **5** | **6** | **7** |
| 11. In general, caries is a serious condition…………………………….. | **1** | **2** | **3** | **4** | **5** | **6** | **7** |
| 12. Caries does not have much effect on a patient’s life………………. | **1** | **2** | **3** | **4** | **5** | **6** | **7** |
| 13. Caries can have serious financial consequences for the patient….. | **1** | **2** | **3** | **4** | **5** | **6** | **7** |
| 14. I have a clear picture or understanding of caries………………….. | **1** | **2** | **3** | **4** | **5** | **6** | **7** |
| 15. The symptoms of caries are puzzling to me……………………….. | **1** | **2** | **3** | **4** | **5** | **6** | **7** |
| 16. I get depressed when I think about patients suffering from caries | **1** | **2** | **3** | **4** | **5** | **6** | **7** |
| 17. Seeing patients with caries does not worry me……………………. | **1** | **2** | **3** | **4** | **5** | **6** | **7** |
| 18. Seeing patients with caries makes me feel angry………………….. | **1** | **2** | **3** | **4** | **5** | **6** | **7** |
| 19. Seeing patients with caries can affect me emotionally (e.g. can make me feel helpless or distressed) ……………………………….. | **1** | **2** | **3** | **4** | **5** | **6** | **7** |

20. *Approximately* how long would it take for a white spot caries in the approximal surface of a molar to progress to the inner half of the dentine in the following circumstances:

a) For a ‘typical’ child under 17 years of age? *(Please circle your choice)*:

**i) Less than 3 mths ii) 3 to up to 6 mths iii) 6 mths to up to 1 yr iv) 1yr to 2 yrs v) More than 2 years**

b) For a child under 17 you classify as high risk? *(Please circle your choice)*:

**i) Less than 3 mths ii) 3 to up to 6 mths iii) 6 mths to up to 1 yr iv) 1yr to 2 yrs v) More than 2 years**

**SECTION 3: fissure sealantS**

|  | *True* | *False* | *Not sure* |
| --- | --- | --- | --- |
| 1a) Fissure sealants are recommended for routine use with high-risk children…………. | **1** | **2** | **3** |
| b) Fissure sealants are appropriate even when small dentine lesions are present………. | **1** | **2** | **3** |
| c) The evidence suggests that resin based sealants are normally preferable to glass ionomer sealants…………………………………………………………………………….. | **1** | **2** | **3** |
| d) The most powerful single predictor of future caries increment in children is socio-economic group…………………………………………………………………………….. | **1** | **2** | **3** |
| e) Once applied, fissure sealants only need attention if problems are encountered | **1** | **2** | **3** |

2. *Approximately* how long, on average, does a fissure sealant last? ____________________________

**SECTION 4: Deciding to place a fissure sealant**

**The following scenarios include various elements that may influence your decision to place a fissure sealant. We appreciate that the scenario format means that skills you may normally draw on (e.g. non-verbal aspects) cannot be a factor in your assessment. Nevertheless, we ask you to address *each* scenario and make a decision as to whether you would or would not place at least one fissure sealant. We have left a space for you to comment on any aspect of a scenario, or your decision, *if you so choose*.**

| 1. | This 11 year old girl is a poor attender, remains very fearful of treatment, but has only needed one small filling so far. That was 18 months ago, and treatment was very difficult because of the child’s distress. Her circumstances suggest she is at moderate risk of caries in the future. She has come today for a check up. No treatment is needed currently. The clinic is very busy. | | | | | | |
| --- | --- | --- | --- | --- | --- | --- | --- |
|  | **Place a fissure sealant?** | Yes |  | No |  |  |  |

# On the scale 1 to 10, how difficult was it for you to make a decision for this scenario?

| ***Not at all*** | **0** | **1** | **2** | **3** | **4** | **5** | **6** | **7** | **8** | **9** | **10** | ***Extremely Difficult*** |
| --- | --- | --- | --- | --- | --- | --- | --- | --- | --- | --- | --- | --- |

| *If you wish to comment on this decision please do so here.* |  |
| --- | --- |

| 2. | The next patient is a 7 year old girl who is new to the practice. Her 6s are fully erupted and sound, but have deep fissures. Her mother says she has good brushing habits and uses a fluoride paste, but does consume more sweets than she probably should. The child is very nervous, and you find it quite difficult to examine her, particularly because she is quite small. | | | | | | |
| --- | --- | --- | --- | --- | --- | --- | --- |
|  | **Place a fissure sealant?** | Yes |  | No |  |  |  |

# On the scale 1 to 10, how difficult was it for you to make a decision for this scenario?

| ***Not at all*** | **0** | **1** | **2** | **3** | **4** | **5** | **6** | **7** | **8** | **9** | **10** | ***Extremely Difficult*** |
| --- | --- | --- | --- | --- | --- | --- | --- | --- | --- | --- | --- | --- |

| *If you wish to comment on this decision please do so here.* |  |
| --- | --- |

| 3. | The next patient has attended with both parents, who ask you if fissure sealants are a good idea for their son, now aged 13. He had caries in one deciduous tooth, but currently has all 8 molars, which are sound. On examination it is clear that brushing could be better, and his diet includes a lot of sugary foods. | | | | | | |
| --- | --- | --- | --- | --- | --- | --- | --- |
|  | **Place a fissure sealant?** | Yes |  | No |  |  |  |

# On the scale 1 to 10, how difficult was it for you to make a decision for this scenario?

| ***Not at all*** | **0** | **1** | **2** | **3** | **4** | **5** | **6** | **7** | **8** | **9** | **10** | ***Extremely Difficult*** |
| --- | --- | --- | --- | --- | --- | --- | --- | --- | --- | --- | --- | --- |

| *If you wish to comment on this decision please do so here.* |  |
| --- | --- |

| 4. | The next patient is an 15 year old boy who you have seen regularly since age five. He has had two small fillings. Both 6’s and 7’s are heavily stained, but the bitewings show no evidence of caries. You note that when his 6’s erupted you recommended fluoride tablets, but he admitted that he didn’t take them regularly. He says he brushes twice a day, doesn’t eat many sweets but has quite a lot of fizzy drinks. | | | | | | |
| --- | --- | --- | --- | --- | --- | --- | --- |
|  | **Place a fissure sealant?** | Yes |  | No |  |  |  |

# On the scale 1 to 10, how difficult was it for you to make a decision for this scenario?

| ***Not at all*** | **0** | **1** | **2** | **3** | **4** | **5** | **6** | **7** | **8** | **9** | **10** | ***Extremely Difficult*** |
| --- | --- | --- | --- | --- | --- | --- | --- | --- | --- | --- | --- | --- |

| *If you wish to comment on this decision please do so here.* |  |
| --- | --- |

| 5. | This 8 year old patient has an immaculate mouth, has never missed a check-up, and loves to tell you about how good she has been in cleaning her teeth. As always, she has come in with her mother, who asks you to place fissure sealants because her neighbour’s child has just got her teeth done. | | | | | | |
| --- | --- | --- | --- | --- | --- | --- | --- |
|  | **Place a fissure sealant?** | Yes |  | No |  |  |  |

# On the scale 1 to 10, how difficult was it for you to make a decision for this scenario?

| ***Not at all*** | **0** | **1** | **2** | **3** | **4** | **5** | **6** | **7** | **8** | **9** | **10** | ***Extremely Difficult*** |
| --- | --- | --- | --- | --- | --- | --- | --- | --- | --- | --- | --- | --- |

| *If you wish to comment on this decision please do so here.* |  |
| --- | --- |

| 6. | This patient has had four fillings by age 8, has a poor diet, and tends to miss check-up appointments. She is now 11. Her parents and older brother all have poor dental health, oral hygiene and diet, and the family are on a low income. There are now signs of small carious lesions on her 6’s but not on her newly erupted 7s. Your previous 2 attempts to fissure seal the 6’s were unsuccessful, with the sealants coming off very quickly. | | | | | | |
| --- | --- | --- | --- | --- | --- | --- | --- |
|  | **Place a fissure sealant?** | Yes |  | No |  |  |  |

# On the scale 1 to 10, how difficult was it for you to make a decision for this scenario?

| ***Not at all*** | **0** | **1** | **2** | **3** | **4** | **5** | **6** | **7** | **8** | **9** | **10** | ***Extremely Difficult*** |
| --- | --- | --- | --- | --- | --- | --- | --- | --- | --- | --- | --- | --- |

| *If you wish to comment on this decision please do so here.* |  |
| --- | --- |

## Section 5: your general style

| **In general, how true are the following statements about you?** | | ***Not at all true*** | ***Barely***  ***true*** | ***Moderately true*** | ***Exactly***  ***true*** |
| --- | --- | --- | --- | --- | --- |
| **1.** | I can always manage to solve difficult problems if I try hard enough… | **1** | **2** | **3** | **4** |
| **2.** | If someone opposes me, I can find means and ways to get what I want………………………………………………………………………..… | **1** | **2** | **3** | **4** |
| **3.** | It is easy for me to stick to my aims and accomplish my goals………… | **1** | **2** | **3** | **4** |
| **4.** | I am confident that I could deal efficiently with unexpected events.. …. | **1** | **2** | **3** | **4** |
| **5.** | Thanks to my resourcefulness, I know how to handle unforeseen situations…………………………………………………………………….. | **1** | **2** | **3** | **4** |
| **6.** | I can solve most problems if I invest the necessary effort………………. | **1** | **2** | **3** | **4** |
| **7.** | I can remain calm when facing difficulties because I can rely on my coping abilities……………………………………………………………… | **1** | **2** | **3** | **4** |
| **8.** | When I am confronted with a problem, I can usually find several solutions……………………………………………………………………… | **1** | **2** | **3** | **4** |
| **9.** | If I am in a bind, I can usually think of something to do………………. | **1** | **2** | **3** | **4** |
| **10.** | No matter what comes my way, I’m usually able to handle it………… | **1** | **2** | **3** | **4** |

Please return the completed questionnaire in the enclosed pre-paid envelope.

Thank you for your participation in this study, your contribution is very much appreciated.
